# Protein dynamics at invadopodia control invasion–migration transitions in melanoma cells

**DOI:** 10.1038/s41419-023-05704-4

**Published:** 2023-03-11

**Authors:** Marlène Legrand, Antoine Mousson, Philippe Carl, Léa Rossé, Hélène Justiniano, Jean-Pierre Gies, Daniel Bouvard, Emilie Sick, Denis Dujardin, Philippe Rondé

**Affiliations:** 1 CNRS UMR7021, Migration, Invasion and Microenvironnement, Faculté de Pharmacie, Université de Strasbourg, Illkirch, France; 2 CNRS UMR 5237, Centre de Recherche en Biologie Cellulaire de Montpellier (CRBM), Université de Montpellier, Montpellier, France

**Keywords:** Melanoma, Invadopodia

## Abstract

Cell invasion is a highly complex process that requires the coordination of cell migration and degradation of the extracellular matrix. In melanoma cells, as in many highly invasive cancer cell types these processes are driven by the regulated formation of adhesives structures such as focal adhesions and invasive structures like invadopodia. Structurally, focal adhesion and invadopodia are quite distinct, yet they share many protein constituents. However, quantitative understanding of the interaction of invadopodia with focal adhesion is lacking, and how invadopodia turn-over is associated with invasion-migration transition cycles remains unknown. In this study, we investigated the role of Pyk2, cortactin and Tks5 in invadopodia turnover and their relation with focal adhesions. We found that active Pyk2 and cortactin are localised at both focal adhesions and invadopodia. At invadopodia, localisation of active Pyk2 is correlated with ECM degradation. During invadopodia disassembly, Pyk2 and cortactin but not Tks5 are often relocated at nearby nascent adhesions. We also show that during ECM degradation, cell migration is reduced which is likely related to the sharing of common molecules within the two structures. Finally, we found that the dual FAK/Pyk2 inhibitor PF-431396 inhibits both focal adhesion and invadopodia activities thereby reducing both migration and ECM degradation.

## Introduction

Melanoma is a very aggressive form of skin cancer and patients with spreading melanoma have a very poor prognosis with a 5-year survival rate <5% [1]. In melanoma as in many invasive epithelial cancers, such as prostate, head and neck or breast cancer, cancer cells acquire the ability to breach basement membrane using supramolecular complexes called invadopodia. Invadopodia are sub-cortical protrusions that localise matrix-degrading activity to cell-substrate contact points and represent major hubs where many signalling pathways converge [2]. Invadopodia are composed of an actin-rich core which include actin activators, nucleators and regulators that is surrounded by adhesion, scaffolding and signalling proteins [3]. These structures are enriched in proteolytic enzymes such as matrix metalloproteinases (MMPs), which mediate extracellular matrix (ECM) degradation. Significant efforts have been made to characterise the mechanism of invadopodia formation and the sequence of events that allows the correct assembly and regulation of invadopodia components start to be elucidated [4].

However, one of the key questions that remains to be solved concerns the maturation step which regulates the switch between immature invadopodia devoid of invasive properties to fully mature invadopodia able to degrade the ECM. Several proteins or signalling pathways have been already implicated in this process such as Tks5 [5], NHE-1 [6] profilin 1 [7], talin2 [8], Mena^IV^ [9], protrudin [10] or the CCL7/CCR3 axis [11]. In melanoma the transition from in situ melanoma (melanoma cells located in their original tissue) to invasive melanoma seems to be correlated with direct contact of melanoma cells with distal keratinocytes of the epidermal layer thereby triggering Notch signalling pathway [12]. Of note, inhibition of Notch signalling or suppression of cell–cell contact reduced Mena^IV^ expression in tumour cells and invadopodia activity [13] raising the possibility that in situ melanoma display already invadopodia structure that mature during their transition into invasive melanoma.

Another aspect of the invadopodia lifetime that is poorly understood concerns the disassembly process. Several lines of evidence seem to indicate that focal adhesion (FA) and invadopodia turn-over are regulated by similar pathways. Thus, calpain has been linked to focal adhesion and invadopodia disassembly through cleavage of their respective substrates, including talin [14], paxillin [15] and cortactin [16]. In line with this, calpain has also been reported to induce podosome disassembly in dendritic cells, through cleavage of important components like WASP, Pyk2 and talin [17]. FA turn-over which tune the migration speed [18, 19] is also controlled in part by phosphorylation and dephosphorylation processes of key molecules, such as FAK and paxillin [20–22]. While FAK is absent from dot-like invadopodia [23–25], it is interesting to note that RhoG modulate the phosphorylation of paxillin during invadopodia disassembly [26]. Similarly, in Rous sarcoma virus-transformed baby hamster kidney cells, paxillin phosphorylations on tyrosine 31 and 118, also allows invadopodia disassembly [27].

In addition, the turn-over of adhesion structures is controlled by the p21 Rho family of small GTPase. For example, Rac1 induces the turnover of both focal adhesions and podosomes [28, 29]. Controlled focal adhesion turn-over take place notably at the lamellipodia which extend protrusions that mediate cell migration. Recently, Rac1 has also been shown to control invadopodia disassembly via activation of the downstream effectors p21 activated kinase 1 (Pak1) with subsequent phosphorylation of cortactin [30]. Because Rac1 is necessary for both focal adhesion and invadopodia turn-over and focal adhesion turn-over is essential for lamellipodia extension, it is generally admitted that invadopodia assembly and cell migration occurs in a sequential manner. Indeed, this is consistent with the fact that degradation activity at specific areas requires the time necessary for invadopodia assembly and maturation which is difficult to reconcile with sustained migration. Thus, a spatio temporal control of FA and invadopodia turn-over is necessary for proper invasion capabilities. Recently it was shown that this regulation could be made by differential activation of downstream effectors of Rac1. Indeed, local activation of Rac1 at the leading edge promotes Wave2-dependent signalling for further lamellipodia extension [31]. This extension is also under the control of the underneath focal complex turnover driven in part by Dock180 a GEF for Rac1 [20]. On the other hand, Rac1 through activation of another GEF Trio, promotes Pak1-mediated invadopodia disassembly [30].

In this study, using high-end imaging techniques, we investigated invadopodia maturation and disassembly processes. We show that Pyk2 and cortactin are major components of both focal adhesions and invadopodia. During invadopodia disassembly, Pyk2 and cortactin are repeatedly relocated to nearby focal complexes promoting lamellipodia extension. We also found that, during invadopodia turn-over, cell migration is reduced that may be due to the dynamic redistribution of invadopodia components to focal adhesions in close proximity. Finally, we show that a small Pyk2 inhibitor reduced melanoma migration without enhancing invadopodia activity contrarily to a small FAK inhibitor that reduced only the migration process but enhanced matrix degradation via relocation of FA component to invadopodia [24].

## Materials and methods

### Antibodies

For immunofluorescence, anti-cortactin (05-180) from Millipore; anti-paxillin (AHO0492) and mouse anti-phospho-Y402 Pyk2 (44-625G) from Invitrogen; rabbit anti-phospho-Y402 Pyk2 (3291) from Cell Signalling; phalloidin 568 (A12380), phalloidin 647 (A22287) and Alexa Fluor–conjugated secondary antibodies from Molecular Probes.

### Cells culture

Human melanoma cell lines, were purchased from Rockland and cultured in MCDB153/L-15 medium (M7403, Sigma; 11415064, Invitrogen) supplemented with 2% fetal bovine serum, 1.68 mM CaCl_2_ and 1% penicillin/streptomycin. The A375 melanoma cell line from ATCC was maintained in DMEM (11960044, Lonza) supplemented with 10% fetal bovine serum, 1 mM L-glutamine and 1% penicillin/streptomycin antibiotics. Melanoma cells were used between passage 5 and 30.

### Cell transfections

FAK-GFP, Src-Y530F-mCherry were obtained as previously described [32]. Plasmid expressing vinculin-mCherry, Pyk2-GFP, Tks5-GFP and Cortactin-dsRed were kindly provided by I. Lavelin (Weizmann Institute of Science, Rehovot, Israel), J.A. Girault (Institut du fer à Moulin, Paris), S. Courtneige (Oregon Health & Science University, Portland) and M.A. McNiven, (Mayo Cancer Center, Rochester). All plasmids were verified by sequencing and isolated using the JetStarPlasmid kit (Genomed) following the manufacturer’s protocol. Melanoma cells were transfected with 2 µg of vinculin-mCherry or Pyk2-GFP or Tks5-GFP or Cortactin-dsRed using lipofectamine 2000 (Invitrogen), according to the manufacturer instructions. Transfected cells were incubated for 24 h at 37 °C before use.

### Matrix degradation assay and immunofluorescence

Glass bottom Ibidi dish (81158, Ibidi) were coated with fluorescent-labelled-FITC gelatine or Cy3 gelatine as described previously [25]. Then, 3.5 × 10^4^ previously starved melanoma cells were plated on fluorescent gelatine and incubated at 37 °C for 24 h in medium supplemented with 10% fetal bovine serum with or without PF-573228 or PF-431396. Cells were fixed using 4% paraformaldehyde permeabilised using triton-X-100 at 0.1% and incubated in 2% of bovine serum albumin at room temperature. Cells were then labelled for 1 h with anti-cortactin (1/250), anti-phospho-Y402 Pyk2 (1/100), anti-FAK (1/250), anti-phospho-Y118 paxillin (1/250) or anti-paxillin (1/250). After three washes with PBS, cells were incubated with Alexa-488-conjugated goat anti-rabbit (1/1000), Alexa-647-conjugated goat anti-rabbit (1/1000), Alexa-405-conjugated goat anti-mouse (1/1000), Alexa-647-conjugated goat anti-mouse (1/1000), Alexa-568-conjugated phalloidin (1/250) or Alexa-647-conjugated phalloidin (1/250) for 1 h, washed and mounted in Prolong Gold mounting media (Invitrogen). Cells were imaged using Leica TSC SPE confocal microscope (×63 HCX Pl Apo 1.40 NA or ×20 HCX Pl Apo 0.7 NA objective, Wetzlar, Germany). Invadopodia were identified as actin and cortactin rich punctate structure. Areas of degradation were identified as “black holes” within the fluorescent gelatine. Invadopodia and areas of degradations were quantified using ImageJ software. Degradation areas measurements were based on cells displaying degradation activity, and the frequency of degradation was based on randomly selected cells.

### Wound healing and 2,5D invadopodia assay

Melanoma cells were seeded in 12-well plates containing medium supplemented with 10% fetal bovine serum and transfected with siCtrl or siFAK as described before. After 24 h, confluent cell layers were treated with mitomycin C and with PF-573228 or PF-431396 as described before and then wounded with a pipet tip. Images of cell migration were acquired using a Leica DMIRE2 microscope (×10 N PLAN 0.25 NA objective) equipped with a 37 °C 5% CO_2_ control system (Life Imaging Services) with a Leica DC350FX CCD camera piloted by the FW4000 software (1 image every 30 min during 24 h). The 2,5D invadopodia assay was performed as previously described (Mousson). Briefly, melanoma cells were added in the upper side of the insert at 5 × 10^4^ cells and incubated 24 h at 37 °C in 5% CO_2_ incubator. After incubation, inserts were washed with PBS and fixed as described in “Matrix degradation assay and immunofluorescence” section. Cells were imaged using a Leica confocal microscope TSC SPE (#63 HCX Pl Apo 1.40 NA objective, Wetzlar, Germany). 3D images were reconstructed using ImageJ or Imaris. The length of each protrusion containing actin and cortactin staining was calculated using ImageJ.

### Live cell imaging, FRAP and TIRF experiments

For live cell imaging, cells were plated on fluorescent gelatin-coated coverslips and mounted in a Ludin Chamber (Life Imaging Services). The cells were then placed at 37 °C, 5% CO_2_ on a iMIC microscope equipped with a multi-LED Lumencor Spectra X. Images were acquired with an Olympus ×60 TIRFM (1.45 NA) objective every 5–10 min during 24 h and a Hamamatsu Flash 4 V2+ camera (Iwata) piloted by the Live Acquisition software (Till Photonics). Expressing cells were initially located via the GFP or mCherry signal, and were subsequently followed only via dual phase contrast/fluorescent signal together with the FITC or RITC-coupled gelatin substrate. Ten to 20 different fields were sequentially recorded during each experiment using a Marzhauser automated stage piloted by the iMIC software. Mean fluorescence intensity at focal adhesion, invadopodia and underlying substrate at each time points were calculated.

TIRF images were acquired using an iMIC microscope (Till Photonics) equipped with a Cobolt Dual Calypso Laser 491/532 nm and an Olympus 60× TIRFM (1.45 NA) objective. During acquisition, cells plated on plasma-cleaned coverslips, were maintained at 37 °C in a 5% CO_2_ humidified atmosphere using an environmental control system (Life Imaging Services). Azimuthal TIRF images were acquired every 1 min during 1 h on an Hamamatsu Flash 4 V2+ camera (Iwata) and analysed using ImageJ software.

FRAP experiments were done at 37 °C, 5% CO_2_ on a iMIC microscope. To avoid possible artefacts of overexpression, only cells expressing low but detectable amounts of protein were chosen for further analysis. Briefly, fluorescence intensity was measured at low laser power before bleach in selected regions of interest at invadopodia. Photobleaching was done at 100% of the 491 nm line with 4 iterations. Recovery was followed with low laser power at various time intervals until the intensity had reached a steady plateau. For each time point, the intensity of the bleached area was normalised to that of a corresponding unbleached area. Fluorescence during recovery was normalised to the prebleached intensity. FRAP measurements were fitted to a single exponential curve, *I*(*t*) = *I*(0) + *k*1e^−*k*2*t*^ to determine the characteristic time of recovery.

### Statistical analysis

Statistical significance was calculated using Student’s *t* test or ANOVA followed by Tukey’s test for multiple comparisons. Values were considered statistically significant when *P* value was <0.05. For all figures: *P < 0.05; **P < 0.01; ***P < 0.001.

## Results

### Actin and Cortactin labelled both active and inactive invadopodia

**Fig. 1:**
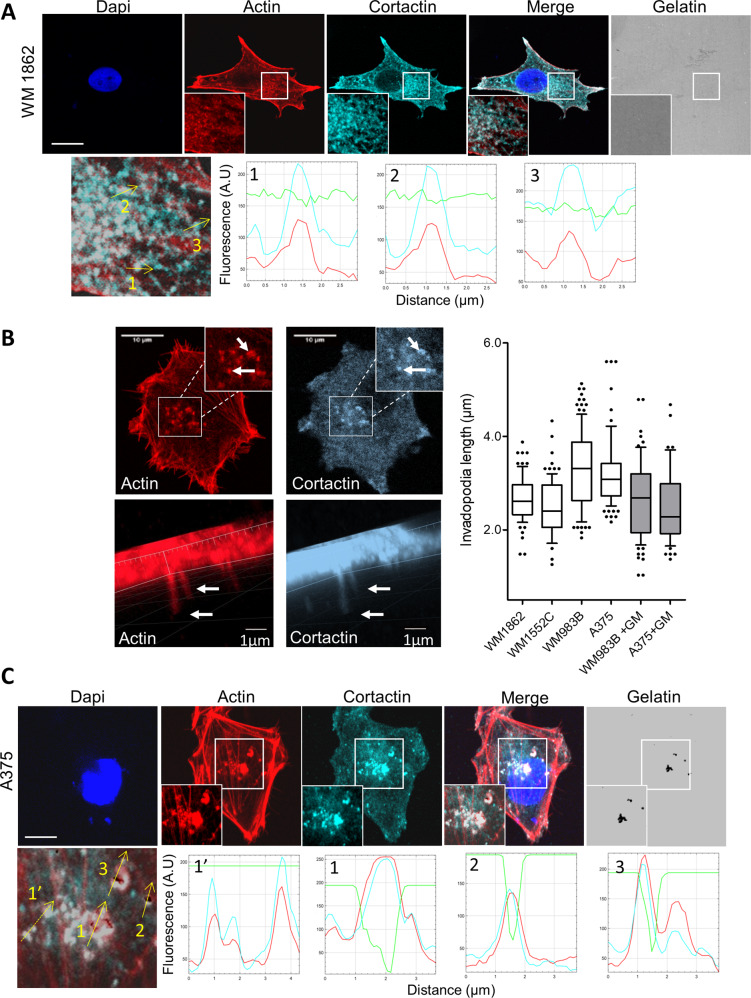
Actin and Cortactin labelled both active and inactive invadopodia. **A** In situ melanoma WM1832 cells were plated on FITC-Gelatin (Grey), fixed, stained with Hoechst and labelled for actin (Red) and cortactin (Cyan). Boxed regions and insets depict invadopodia. Graphs indicate fluorescent intensity in arbitrary units (A.U.) of F-actin (red), Cortactin (Cyan) with respect to Gelatin (green) over the indicated line scan in the inset. Note the colocalisation of actin and cortactin in dot-like structure and the absence of degradation area. Scale bar: 10 µm. **B** *X*–*Y* (top, left) confocal images of in situ melanoma WM1552 showing invadopodia (insets, white arrows) as identified by dot-like F-actin (Red) and cortactin (Cyan) colocalisation. *X*–*Z* (bottom, left) images of invadopodia projecting into a collagen/gelatin matrix plated in the top chamber of a 1-μm transwell filter. Histograms represents mean invadopodia length and whiskers with 10–90 percentile with 54–117 invadopodia from 27 to 56 cells analysed per condition. **C** Metastatic melanoma A375 cells were plated on FITC-Gelatin (Grey), fixed, stained with Hoechst and labelled for actin (Red) and cortactin (Cyan). Boxed regions and insets depict invadopodia (White) and degradation areas were identified as black holes on fluorescent gelatin. Graphs indicate fluorescent intensity in arbitrary units (A.U.) of Actin (red), Cortactin (Cyan) with respect to Gelatin (green) over the indicated line scan in the inset. Dotted line indicates inactive invadopodia whereas straight lines indicate active invadopodia. Note that co-localisation of actin and cortactin in dot-like structure label both active and inactive invadopodia. Scale bar: 10 µm. The mean number of invadopodia/cell was calculated for 57 WM1862 and 123 A375 cells.

To elucidate the specific role of invadopodia-related protein during invadopodia maturation, we first plated melanoma cells on fluorescent gelatin and analysed invadopodia activity. We recently reported that melanoma cells form inactive invadopodia when located in the epidermis that matures into fully degrading invadopodia during the transition from in situ to metastatic locations [25]. Indeed, we found that, although many invadopodia, identified as actin- and cortactin-rich punctate structures, were present in in situ WM1862 melanoma cells (mean value: 13.87 ± 1.19 invadopodia/cell) none of them displayed degradative properties as reported by in situ zymography assay (Fig. 1A, see quantification of the gelatin signal in green along the yellow arrows 1, 2 and 3). To confirm the identity of these actin- and cortactin-rich structures we tested their capacity to form ventral protrusions when plated on 1 µm transwell filters. 3D reconstruction of confocal images indicated the presence of ventral protrusions containing actin and cortactin in both in situ (WM 1862 and WM 1552 C) and malignant cell lines (WM983B and A375). Of note invasive melanoma displayed longer invadopodia compared to non-invasive cells that was reduced in the presence of the metalloprotease inhibitor GM6001 (Fig. 1B).

We next analysed invadopodia activity in metastatic A375 melanoma cells. As previously reported, many invadopodia were identified in these cells using dot-like actin/cortactin colocalisation (mean value: 14.34 ± 0.57 invadopodia/cell) but only a few of them were able to degrade the fluorescent gelatin, the degradation frequency being 20.08% ± 1.81 (Fig. 1C). Moreover, invadopodia-induced gelatin degradation was completely inhibited in the presence of GM6001 (Supplemental Fig. 1). Thus altogether, these results indicate that actin and cortactin labelled both active and inactive invadopodia.

### Pyk2 localisation at invadopodia correlates with matrix degradation

**Fig. 2:**
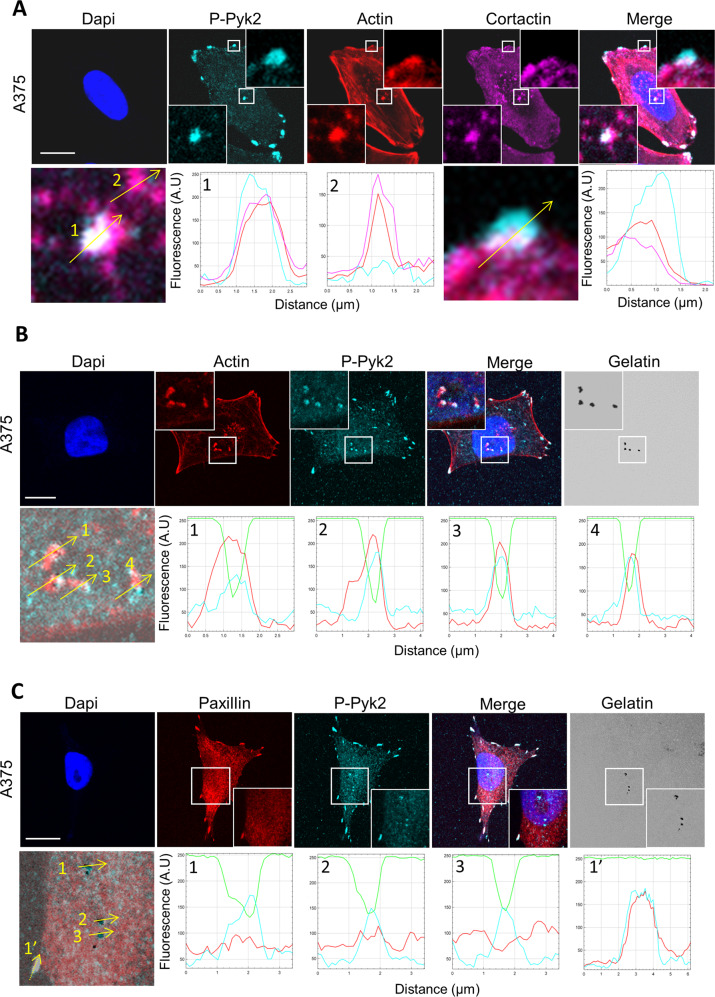
P-Pyk2 is expressed at both focal adhesion and invadopodia. **A** Metastatic melanoma A375 melanoma cells were fixed stained with Dapi and labelled for P-Pyk2 (Cyan) actin (Red) and cortactin (Magenta). Boxed regions and insets depict focal adhesion (top, white) and typical dot-like invadopodia (bottom white). Graphs indicate fluorescent intensity in arbitrary units (A.U.) of P-Pyk2 (Cyan), actin (red) and cortactin (Magenta) over the indicated line scan in the inset at invadopodia (left) and focal adhesion (right). Note high level of P-Pyk2 in only one of the two invadopodia selected. Note also high level of P-Pyk2 at focal adhesion. Scale bar: 10 µm. **B** Metastatic melanoma A375 cells were plated on FITC-Gelatin (Grey), fixed, stained with Hoechst and labelled for actin (Red) and P-Pyk2 (Cyan). Boxed regions and insets depict invadopodia (White) and degradation areas were identified as black holes on fluorescent gelatin. Graphs indicate fluorescent intensity in arbitrary units (A.U.) of actin (red), P-Pyk2 (Cyan) with respect to gelatin (green) over the indicated line scan in the inset. Note that all active invadopodia present high level of both actin and P-Pyk2. Scale bar: 10 µm. **C** Metastatic melanoma A375 cells were plated on FITC-Gelatin (Grey), fixed, stained with Hoechst and labelled for paxillin (Red) and P-Pyk2 (Cyan). Boxed regions and insets depict invadopodia and focal adhesion (White). Degradation areas were identified as black holes on fluorescent gelatin. Graphs indicate fluorescent intensity in arbitrary units (A.U.) of paxillin (red), P-Pyk2 (Cyan) with respect to gelatin (green) over the indicated line scan in the inset. Note that all active invadopodia (straight lines) present high level of P-Pyk2 but background level of paxillin whereas focal adhesions (dotted line) present high level of both paxillin and P-Pyk2 but no degradation. Scale bar: 10 µm.

**Fig. 3:**
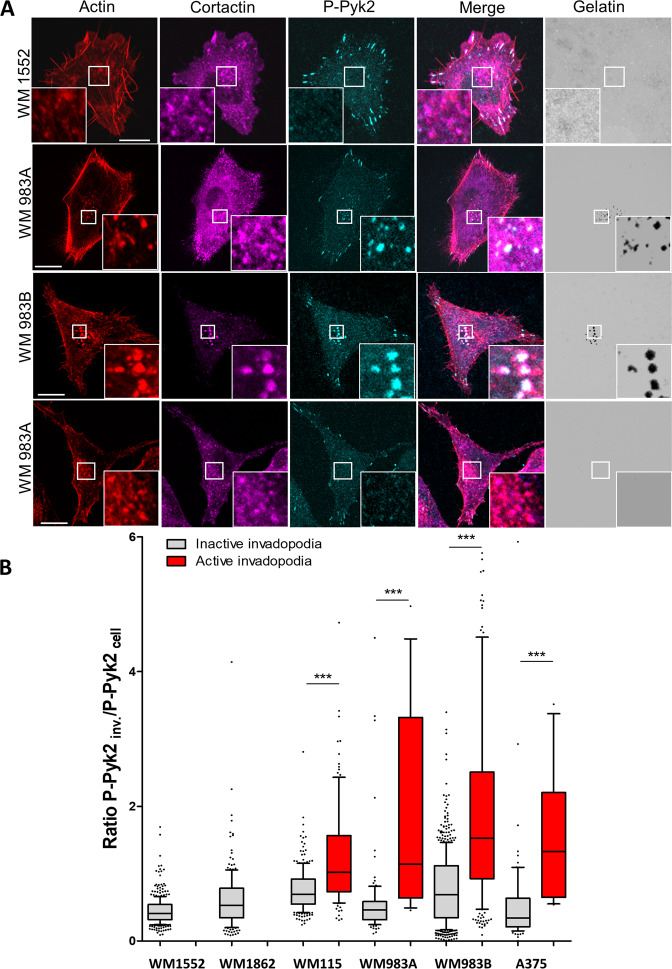
P-Pyk2 localisation at invadopodia correlates with matrix degradation. **A** In situ and metastatic melanoma cells were plated on FITC-Gelatin (Grey), fixed, and labelled for actin (Red) cortactin (Magenta) and P-Pyk2 (Cyan). Boxed regions and insets depict invadopodia (White) and degradation areas were identified as black holes on fluorescent gelatin. Note the presence of P-Pyk2 at focal adhesions but also in a subset of invadopodia from metastatic melanoma cells. Note the presence of “active invadopodia” defined as colocalisation of actin/cortactin spots with degradation areas whereas inactive invadopodia are defined as actin/cortactin spots without underneath degradation. Scale bar: 10 µm. **B** Histograms represents mean and whiskers with 10–90 percentile of the ratio P-Pyk2 signal at invadopodia over P-Pyk2 signal in the whole cell calculated for each active (0–256, in red) and inactive invadopodia (79–569, in grey) from at least 10 cells per cell type from 3 independent experiments; ***P < 0.001; ANOVA followed by Tukey’s Multiple Comparison for active versus inactive conditions.

Recently, Pyk2 was found to localise to invadopodia in invasive breast cancer cells. We thus sought to determine the role of Pyk2 in invasive melanoma cells. Immunofluorescent labelling of endogenous activated Pyk2 (pY402-Pyk2) in A375 cells showed colocalisation with cortactin and actin at both ventral areas and peripheral structures resembling to focal adhesions. Of note, the distribution of activated Pyk2 at ventral areas display a more restricted pattern than the total distribution of invadopodia identified by dot-like actin cortactin co-localisation (Fig. 2A, see quantification of the signal along the yellow arrows 1 and 2). To test whether Pyk2 is located at active invadopodia, A375 cell were plated on fluorescent ECM and colocalisation analysis of Pyk2 with actin spots at degradation puncta was performed. We found consistent localisation of activated Pyk2 only at actin positive structures associated with areas of degradation (Fig. 2B). Next, to clearly identify focal adhesions, immunofluorescent experiments were performed using an anti-paxillin antibody. Activated Pyk2 colocalised with paxillin at focal adhesions whereas paxillin did not colocalised with Pyk2 at ventral degradation areas indicating that Pyk2 contrarily to paxillin localised to both focal adhesions and active invadopodia (Fig. 2C). These results suggest that Pyk2 may be a marker of active invadopodia. To test this hypothesis, immunofluorescent labelling of activated Pyk2 was assessed in a panel of melanoma cells ranging from non-invasive in situ melanoma (WM1552 and WM1862) to invasive (WM115 and WM983A) and metastatic melanoma (WM983B and A375). In in situ melanoma, displaying immature invadopodia unable to degrade the ECM, Pyk2 localisation was restricted to focal adhesions (Fig. 3A). In close opposition, active Pyk2 was observed at both focal adhesions and mature invadopodia, in invasive and metastatic cells. Of note, immature invadopodia present in invasive cells do not display detectable amount of active Pyk2 (Fig. 3A). Quantification of pY402-Pyk2 at active versus inactive invadopodia in this panel of melanoma cells revealed that active Pyk2 is significantly over-expressed only at active invadopodia indicating a major role of Pyk2 in invadopodia maturation processes (Fig. 3B and Supplemental Table 1).

### Pyk2-GFP and Cortactin-dsRed localise to a subset of focal adhesion and invadopodia

To examine the role of these proteins in the stages of invadopodium maturation and disassembly and their potential relation with focal adhesion in more detail, we next used time-lapse videomicroscopy. Previously, we have shown that FAK is not located at invadopodia in melanoma cells [24, 25]. Indeed, in A375 cells expressing FAK-GFP, FAK colocalised with activated Pyk2 and paxillin at focal adhesions whereas Pyk2 but not FAK colocalised with actin dots at invadopodia (Supplemental Fig. 2A). Similarly, in A375 cells expressing vinculin-mCherry, vinculin colocalised with actin and cortactin at focal adhesions but not at invadopodia (Supplemental Fig. 2B). On the other hand, both Pyk2 and cortactin localised to a subset of focal adhesions and invadopodia in invasive melanoma expressing either Pyk2-GFP or cortactin-dsRed. Indeed, in these cells, Pyk2-GFP localised to peripheral focal adhesions and to some invadopodia identified by dot-like actin/cortactin colocalisation (Supplemental Fig. 3A) whereas cortactin-dsRed localised to some focal adhesions and to invadopodia (Supplemental Fig. 3B). This result suggests a dynamic recruitment of Pyk2 at specific stages of invadopodia assembly and further show that cortactin and/or Pyk2 are suitable for invadopodia-focal adhesion interplay investigations.

### Loss of Cortactin at invadopodia is time-correlated to the formation of focal adhesion-containing cortactin during migration

**Fig. 4:**
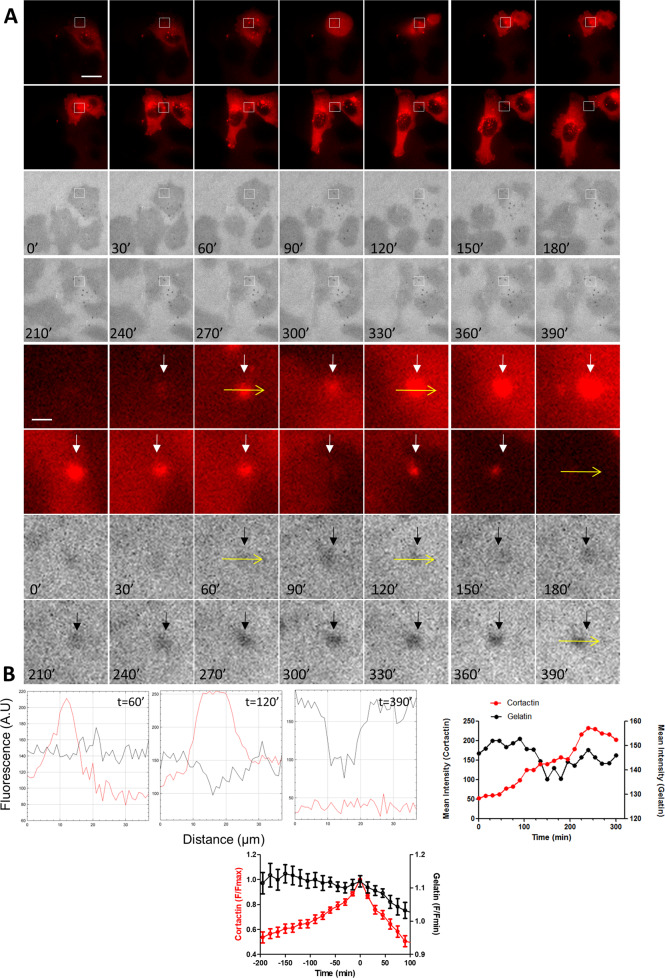
Time-lapse imaging of Cortactin-dsRed revealed biphasic Cortactin appearance at active invadopodia. **A** Dual time-lapse images of Cortactin-dsRed and Cy3-Gelatin were taken every 15 min for 24 h; representative images are shown every 30 min. Insets show magnified views of active invadopodia in the cortactin channel (white arrows) and the gelatin channel (black arrows) Scale bar: 10 µm. **B** Graphs indicate fluorescent intensity in arbitrary units (A.U.) of Cortactin-dsRed (Red), and Cy3-gelatin (black) over the indicated yellow line scan in the inset at *t* = 60, 120 and 390 min. Note high fluorescence intensity of Cortactin-dsRed (left) at invadopodia before degradation (*t* = 60), a second increase in fluorescence intensity after degradation (*t* = 120) and the return to background levels after degradation (*t* = 390). Right graph represents the mean intensity of Cortactin-dsRed and Cy3-gelatin fluorescence over time of this example. Bottom graph represents the mean *F*/*F*_max_ intensity ± SEM of Cortactin-dsRed and *F*/*F*_min_ intensity ± SEM of FITC-gelatin over time from 30 invadopodia. For each invadopodia, *T* = 0 was defined as the maximal fluorescence intensity of cortactin.

**Fig. 5:**
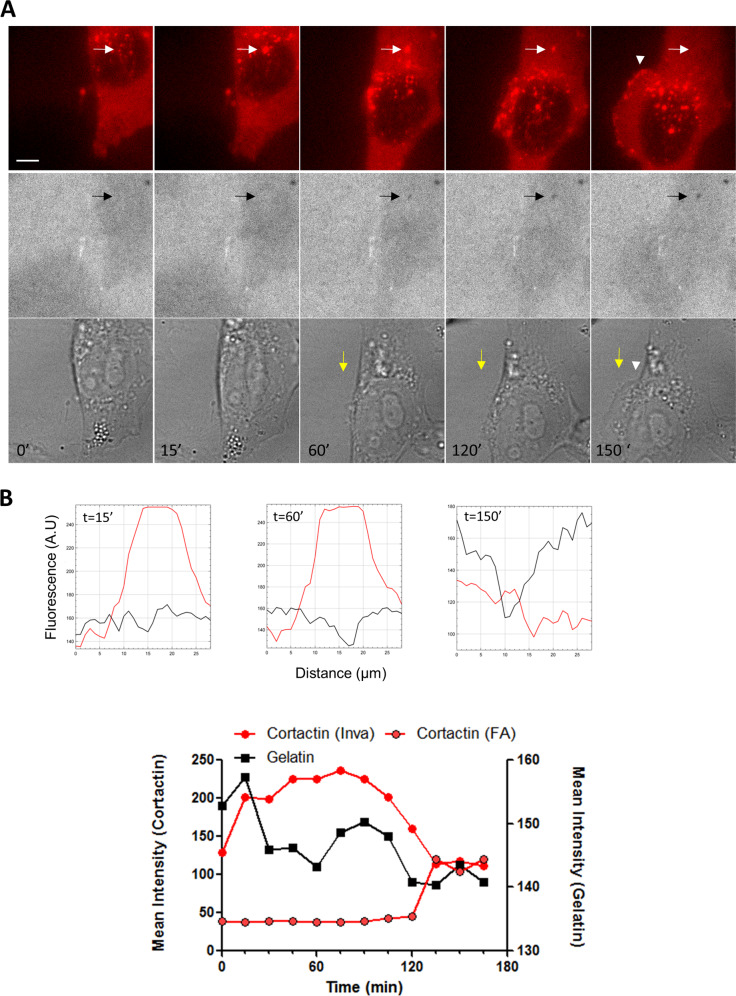
Loss of Cortactin at invadopodia is time-correlated to the formation of focal adhesion-containing Cortactin during migration. **A** Dual fluorescent time-lapse images of Cortactin-dsRed and Cy3-Gelatin and bright field images were taken every 15 min for 24 h; representative images are shown. Dot-like cortactin fluorescence appeared at *t* = 15 min (white arrow) and disappeared at *t* = 150 min, whereas matrix degradation appeared at *t* = 60 min (black arrow). Note that the disappearance of cortactin at invadopodia is time-correlated to the appearance of cortactin at focal adhesion (arrowhead) during migration clearly visualised by the movement of the nucleus in the bright field images (yellow arrow) Scale bar: 10 µm. **B** Graphs indicate fluorescent intensity in arbitrary units (A.U.) of Cortactin-dsRed (Red), and Cy3-gelatin (black) over the indicated line scan in the inset at *t* = 15, 60 and 150 min. Note high fluorescence intensity of Cortactin-dsRed (left) at invadopodia before degradation (*t* = 15), and the return to background levels after degradation (*t* = 150). Graph represents the mean intensity of Cortactin-dsRed at invadopodia and focal adhesion and Cy3-gelatin fluorescence at invadopodia over time. Note the concomitant increase and decrease in cortactin fluorescence at focal adhesion and invadopodia, respectively.

Next, using time-lapse imaging of live A375 cells expressing cortactin-dsRed plated on fluorescent gelatin, we found that cortactin localisation at invadopodia occurs several minutes before gelatin degradation (Fig. 4 and Video 1) in agreement with previous studies [33]. The increase in cortactin level reached a peak about 200-230 min after the beginning of the invadopodia formation with a second phase that seems more pronounced at *t* = 250 (Fig. 4B) which may be related to the established role of cortactin in the regulation of protease secretion [34]. Analysing in further details invadopodia turn-over, we observed, intact, actively sliding invadopodia translocation toward the peripheral cell border as the cell migrate forward which end-up into the complete disassembly of the structure (Fig. 5 and Video 2). It is interesting to note that the disappearance of cortactin at this site is temporally correlated to the appearance of cortactin at nearby nascent adhesion in 71,4% of the cells displaying active invadopodia, suggesting that the formation of these adhesions could be related to the disassembly of invadopodia via exchange of cortactin (Fig. 5A, B).

### Disassembly of invadopodia-containing cortactin and Pyk2 trigger nascent adhesion formation

**Fig. 6:**
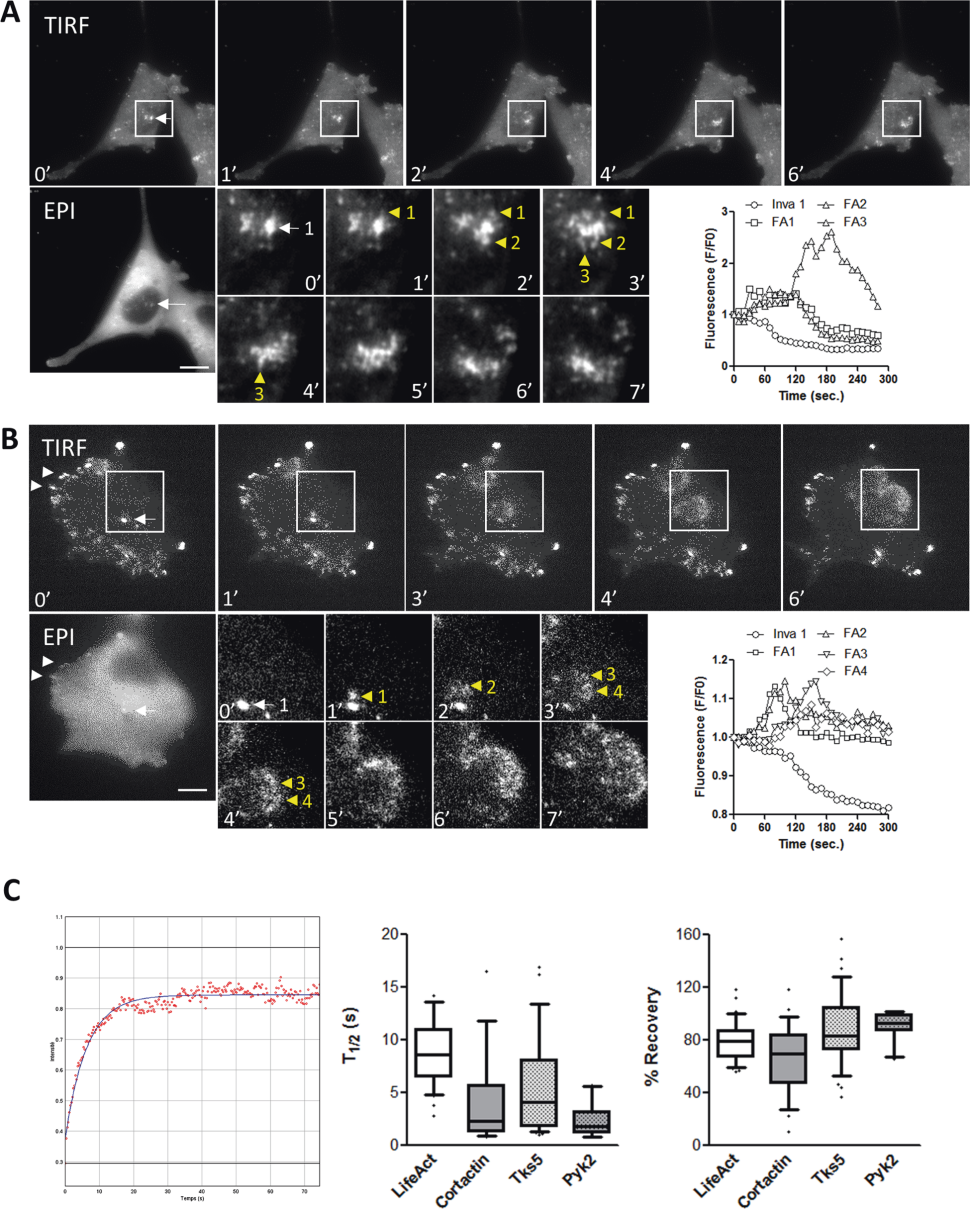
Analysis of invadopodia dynamics and protein dynamics at invadopodia. A375 cells expressing either Cortactin-dsRed (**A**) or Pyk2-GFP (**B**) were imaged by dual Epifluorescence/TIRF mode at 1 image/10 s during 1 h. Insets show magnified views of invadopodia (white arrows). One image in epifluorescence (EPI) is shown for each condition to help visualise the cortical location of invadopodia. Note the transformation of dot-like invadopodia into focal complexes (yellow arrowheads) in cells expressing cortactin (**A**) and Pyk2 (**B**). Representative time-lapse images are shown from 27 to 37 cells. Graph represent normalised fluorescence intensity over time at invadopodia and nascent adhesions. Scale bar: 10 µm. **C** Analysis of protein dynamics at invadopodia by FRAP. Left, a typical recovery curve of Tks5-GFP at invadopodia is shown. Middle, box chart represent the mean *t*1/2 and whiskers the 10–90 percentile from 10 to 35 cells analysed per condition. Right, box chart represent the mean % of recovery and whiskers the 10–90 percentile from 10 to 35 cells analysed per condition.

To investigate protein dynamic at invadopodia at high resolution and analyse the relationship between invadopodia and focal adhesion, we performed dual epifluorescence/TIRF microscopy of A375 cells transfected with either dsRed-cortactin, or Pyk2-GFP. Time-lapse acquisition of dsRed-cortactin revealed several dot-like invadopodia localised at the ventral area of the cells that can be visualised in both epifluorescence and TIRF microscopy (Fig. 6A, left, Video 3). During the time course of the experiment we observed by TIRF microscopy that, in 62.6 % of the cells analysed, many dot-like invadopodia, gradually changed their morphology to adopt more dispersed structures (arrowheads 1–3) resembling to nascent adhesion (Fig. 6A). The time-course of these changes is relatively rapid and occurred in 5–6 min which is consistent with the previously reported time of invadopodia disassembly [30]. To clearly characterise these structures, we then used cells expressing Pyk2-GFP as we have shown that P-Pyk2 is both a marker of active invadopodia and also located at focal adhesion (Figs. 2 and 3). Indeed, we identified both dot-like invadopodia (arrows) at ventral areas and focal adhesion at peripheral areas (arrowheads) with epifluorescence and TIRF microscopy (Fig. 6B, left, Video 4). Time-lapse acquisition in TIRF mode of Pyk2-GFP shows that, in 66.7% of the cells analysed, newly formed fluorescent adhesions were initiated at the dot-like invadopodia location before spreading away in a manner similar to what has been described for nascent adhesion dynamics at lamellipodia (Fig. 6B, insets). We also observed that Pyk2 fluorescence at invadopodia decreases while it increases at nearby nascent adhesion suggesting that invadopodia and focal adhesion likely exchange protein component during their turn-over. To verify whether this mechanism is specific to protein shared by both invadopodia and focal adhesions we performed time-lapse imaging of Tks5-GFP a protein not found in other protrusions and adhesion structures [35–37]. Several Tks5-GFP-containing invadopodia were clearly identified by both epifluorescence and TIRF microscopy (Supplemental Fig. 4 and Video 5). However, in close opposition to the observations made in cells expressing either cortactin-dsRed or Pyk2-GFP, dynamic analysis of Tks5-GFP over time by TIRF microscopy revealed that, translocation of this protein to other structure could be observed in only 11,5 % of the cells analysed. Protein dynamic at invadopodia and their exchange rate with focal adhesion is dependent on the time-residency of these proteins at invadopodia. We thus assessed these parameters by fluorescence recovery after photobleaching (FRAP) experiments. The GFP, ds-Red or m-Cardinal moiety contained in either Tks5, Pyk2, cortactin or actin constructs present at invadopodia randomly selected at the ventral part of the cells were photobleached with short high-power 491 or 532 nm laser excitation, and the recovery of fluorescence in the bleached regions was followed by time-lapse imaging in TIRF mode over the ensuing 200 s, at invadopodia. Recovery of fluorescence after photobleaching at invadopodia was fast for all the 4 proteins investigated indicating that invadopodia components are continuously renewed during their life-time (Fig. 6C). Of note, Pyk2 (*t*1/2 = 2.30 ± 0.48 s) and to a lesser extend cortactin (*t*1/2 = 4.36 ± 1.01 s) are substantially faster compared with Tks5 (*t*1/2 = 5.77 ± 0.90 s) or actin (*t*1/2 = 8.57 ± 0.51 s), which may be related to the dynamic exchange of Pyk2 and cortactin between invadopodia and focal adhesion.

### Migration speed is reduced during invadopodia activity

**Fig. 7:**
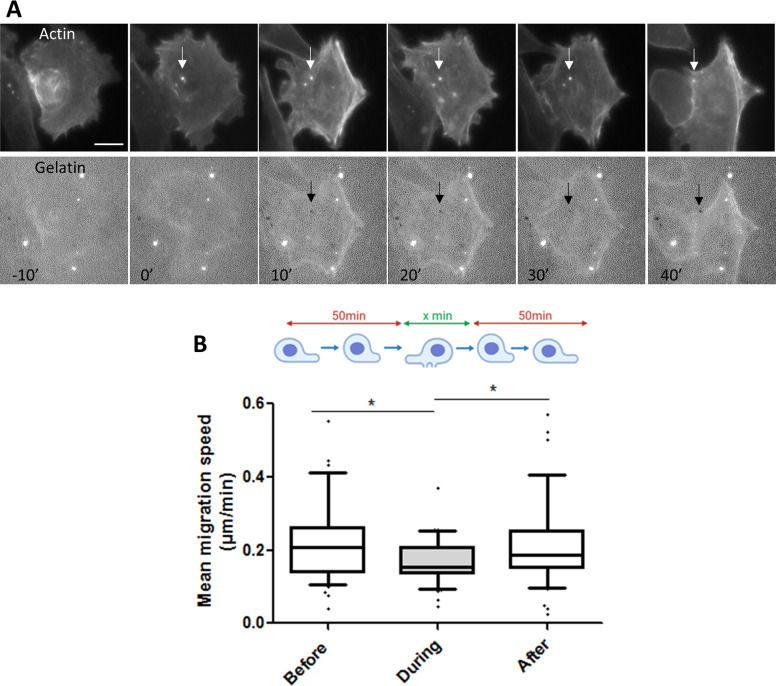
Melanoma displayed deceased migration during invadopodia activity. **A** Dual time-lapse images of LifeAct-mCardinal and FITC-Gelatin were taken every 10 min for 24 h; representative images are shown every 10 min. Note the presence of actin spot (t = 0; white arrow) before the degradation activity (*t* = 10; black arrow) Scale bar: 10 µm. **B** Box chart represent the mean migration speed of cells and whiskers the 10–90 percentile (*n* = 45, from 3 independent experiments) before and during invadopodia activity. *P < 0.05; unpaired *t* test compared to control condition.

Because invadopodia and nascent adhesions exchange proteins essential to maintain their structure we hypothesised that cell migration and active invadopodia assembly occurs sequentially. To test this hypothesis we acquired time-lapse images of A375 metastatic melanoma cells known to spontaneously assemble invadopodia. Cells were engineered to express the fluorescent invadopodia marker mCardinal-LifeAct and cultured on fluorescent gelatin. Cell migration speed were quantified by tracking the nucleus path over time before the invadopodia assembly, during their life-time, and after their disassembly (Fig. 7). In the beginning of the recording, before invadopodia assembly, the tracked cells migrate relatively fast at a mean value of 0.232 ± 0.021 µm/min. However, during the lifetime of active invadopodia the cells migrate significantly less rapidly (0.180 ± 0.013 µm/min) before returning close to the initial migration speed (0.222 ± 0.021 µm/min) after complete invadopodia disassembly (Fig. 7B). This result clearly indicates a negative balance between invadopodia activity and migration. Taken together, our results suggest that the frequency of switching between the invasion state and migration state is, at least in part, controlled by the exchange rate of common proteins at invadopodia and focal adhesions.

### The dual FAK/Pyk2 inhibitor PF-431396 reduced both migration and matrix degradation of melanoma cells

**Fig. 8:**
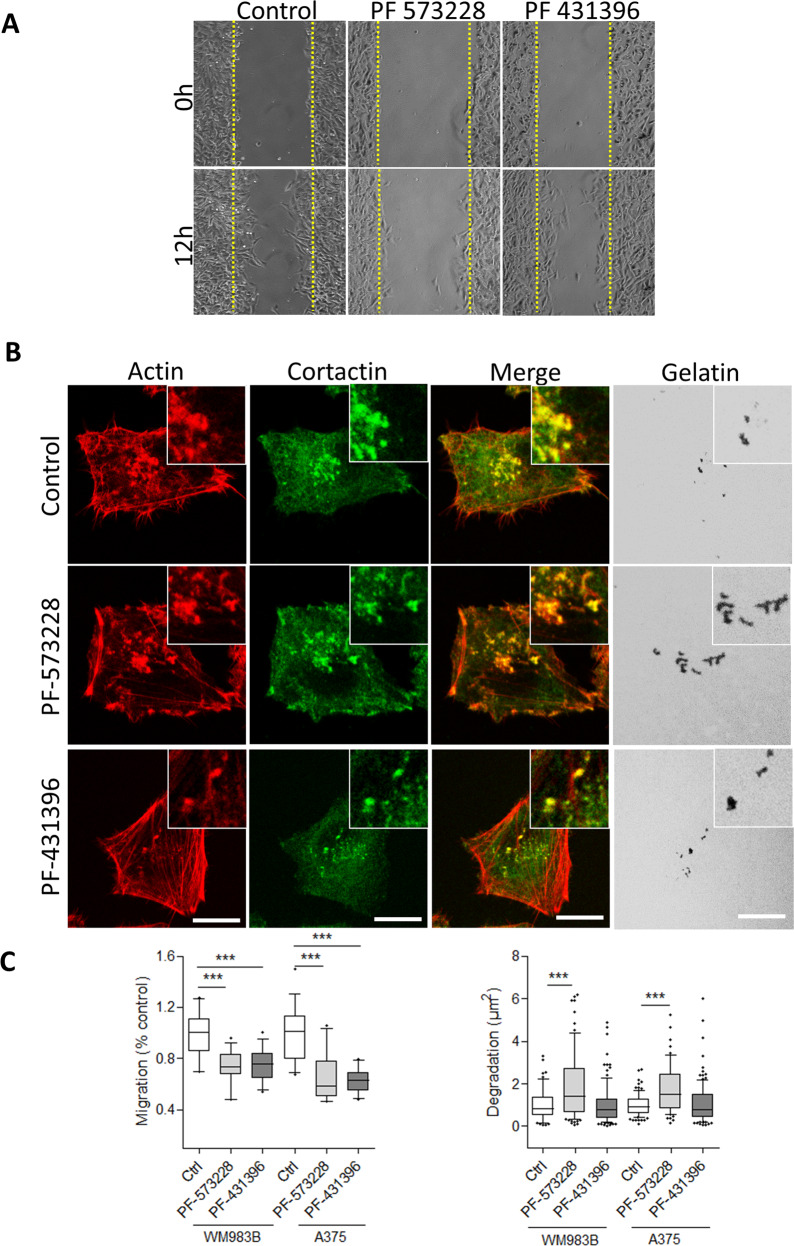
Effect of the FAK inhibitor PF-573228 and the dual FAK/Pyk2 inhibitor PF-431396 on migration and matrix degradation of melanoma cells. **A** Confluent cell layers of A375 cells treated or not with PF-573228 or PF 431396 at 1 µM were wounded and cells were allowed to migrate during 12 h. **B** A375 melanoma cells lines treated or not with PF-573228 or PF 431396 at 1 µM were plated on Cy3-Gelatin (Grey), fixed, and labelled for actin (Red) and cortactin (Green). Scale bar: 10 µm. **C** Left, box chart represent the mean migration speed normalised to control of cells and whiskers the 10–90 percentile from at least 3 independent experiments. Right, box chart represent the mean area of degradation (*n* = 66–118) from 3 independent experiments and whiskers the 10–90 percentile. ***P < 0.001; unpaired *t* test compared to control condition.

Since tumour invasion requires both cell migration and MEC degradation, we investigated whether a pharmacological inhibitor of a protein implicated in both processes would be an efficient strategy to prevent cell invasion. Indeed, several kinase inhibitors targeting migration and/or invasion processes are currently into clinical trials [38–40]. Thus, we compared the effect of the selective FAK inhibitor PF-573228 and the dual FAK/Pyk2 inhibitor PF-431396 on melanoma cell migration and ECM degradation. Using the wound healing assay, we observed that FAK inhibition reduced the migration speed of metastatic melanoma cells by 30–40% compared to control conditions, as already demonstrated in other cancers cell lines (Fig. 8A–C). These results confirmed that FAK expression is necessary for optimal melanoma cell migration independently of melanoma aggressivity. Interestingly, treatment with PF-431396 did not further inhibit cell migration as compared to treatment with PF-573228 suggesting FAK and Pyk2 may have redundant effects on cell migration in these cell lines. We next analysed the effect of FAK inhibition and dual FAK/Pyk2 inhibition on invadopodia properties. Confirming our previous results, we show that FAK inhibition significantly increased invadopodia-induced matrix degradation as compared to control conditions (Fig. 8B, C). In close opposition, dual FAK/Pyk2 inhibition did not modify invadopodia activity. This indicates that Pyk2 inhibition is sufficient to counteract the effect of FAK inhibition-mediated increased invadopodia activity. These results are in line with the localisation of active Pyk2 at invadopodia in these cells shown in Figs. 3 and 4 and confirmed previous data establishing that Pyk2 is necessary for invadopodia activity in breast cancer cell line [41]. Altogether, these results indicate that inhibition of both focal adhesion and invadopodia activity may represent a novel approach to reduce metastatic processes.

## Discussion

Using live-cell video microscopy, high resolution TIRF microscopy and FRAP analysis, we have characterised protein dynamics at invadopodia of melanoma cells adhering to a thin 2D matrix. To our knowledge, we are the first to demonstrate that components shared by invadopodia and focal adhesions, such as Pyk2 and cortactin displayed rapid dynamics at invadopodia allowing fast redistribution to nascent adhesions during invadopodia disassembly.

In this study, we observed invadopodia formation and elongation in in situ and metastatic human melanoma cell lines using 2,5D invasion assays. As invadopodia initially form precursors enriched in F-actin, Arp2/3 and cortactin which then mature to acquire proteolytic activity [5, 42, 43], we suggest that in situ melanoma cells assemble invadopodia precursor and that the maturation step occurs during melanoma progression. In agreement, previous studies described the possible involvement of signalling pathways in invadopodia maturation. Notch signalling has been described to initiate melanoma progression by inhibiting MITF function [12]. Moreover, Notch signalling initiate Mena^IV^ expression which stimulate invadopodia formation and function by enhancing the phosphorylation of Y421 cortactin [9]. Our results show that another protein, Pyk2 is specifically localised at degradative but not immature invadopodia thereby identifying Pyk2 as a major protein implicated in the maturation process. The mechanism implicated in Pyk2-induced invadopodia activity remains to be determine but recent studies have shown that Pyk2 recruits Src kinase into invadopodia and control cortactin phosphorylation in breast cancer cells [41] suggesting a possible mechanism for invadopodia maturation. This mechanism may depend on MMP activity as its inhibition reduced both invadopodia length and Pyk2 localisation to invadopodia as reported here.

Depending of the cells studied, it has been reported that the life-time of invadopodia is relatively high, ranging from minutes to hours thereby allowing significant matrix degradation at these sites. Therefore, for efficient ECM degradation leading to dissemination of melanoma cells, high migration speed should be either avoided or redirected toward the degradation area. We found that, during invadopodia lifetime, melanoma migration speed was reduced confirming previous studies on breast carcinoma [44]. FA are key regulators of migration speed, and our results raise the possibility that signalling cues originating from invadopodia may regulate FA dynamics. Indeed, as FA and invadopodia share many structural components, a dynamic equilibrium of proteins between FA and invadopodia could represent a mean to regulate the cycle between proteolytic ECM remodelling and cell migration thereby enhancing the invasion process. In support to this hypothesis, we observed fast dynamic of Pyk2 and cortactin at invadopodia together with rapid redistribution of these proteins to newly formed nascent adhesions at lamellipodia during invadopodia disassembly. The fact that this redistribution occurred in 62–66% of the cells analysed suggest that theses exchanges of component take place at both immature and mature invadopodia. This dynamic exchange of proteins is in agreement with previous studies showing that Src is located either at invadopodia or at FA depending on the FAK phosphorylation state of the cells, thereby regulating either invadopodia activity or FA dynamics [23, 24]. Although Pyk2 localisation was described as mainly cytosolic, previous studies have found that Pyk2 localised to FA in certain cancer cell lines [45, 46]. Moreover, recently it was shown that cortactin is a substrate and interactor of Pyk2 [41] and our study revealed the presence of cortactin and Pyk2 at both nascent adhesions and invadopodia suggesting that the shuttling of these molecules between the two structures regulate the cycling between migration and ECM degradation.

However, invadopodia are believed to be distinct from FA based on their molecular components, architecture and spatio-temporal regulation. For example, invadopodia contain proteins that regulate actin polymerisation, such as the Arp2/3 complex and N-WASP contrarily to FA. Nevertheless, in Src-transformed fibroblasts, invadopodia formation is initiated at adhesion sites in response to the focal generation of phosphatidylinositol-3,4-bisphosphate that triggers the recruitment of a protein complex including Grb2, Tks5 and N-WASP [47]. Therefore, approaches to identify key molecules that drive the transition between FAs to invadopodia have been used and led to the discovery that the combination of low PKCα activity with increased PI3K promotes enhanced formation and activity of invadopodia along with changes in the organisation of focal adhesions [48]. Furthermore, because adhesion-related protein are often located at or near invadopodia it has become clear that FA may contribute at least in some conditions to the formation of invadopodia [2]. Conversely, our study shows that invadopodia also may contribute to the formation of nascent adhesions. This is in agreement with previous observations showing that, in Src-transformed fibroblasts, rapid rosette disassembly was frequently accompanied by localised, dynamic bursts of lamellipodia and nascent focal adhesions formation [49]. Thus, we propose the existence of a dynamic exchange of protein components between these two adhesion structures aimed at controlling the cycles of migration and ECM degradation for efficient invasion.

Our findings indicate that activated Pyk2 may be a marker of melanoma aggressiveness as its expression was only detected in invasive melanoma. This confirmed a recent study showing that increased expressions of P-Pyk2 were positively correlated with advanced stage, metastasis, and Clark grade in melanoma patients [50]. Moreover, altogether our results suggest also the interesting possibility of targeting protein components shared by both invadopodia and focal adhesions to reduce invasion processes. To challenge this hypothesis we tested the dual FAK/Pyk2 inhibitor PF-431396 on ECM degradation and migration assays. We found that, contrarily to the selective FAK inhibitor PF-573228, which reduced cell migration but enhanced ECM degradation, PF-431396 significantly inhibited cell migration without altering ECM degradation. We and others have already shown that inhibiting FAK via either pharmacological inhibitors targeting the kinase domain, siRNA strategies or FAK mutation at Y397, increases ECM degradation via inhibition of the Src binding domain at FA [23, 24] thereby enhancing Src localisation to invadopodia targets. Of note, among invadopodia targets, Pyk2 was recently shown to interact with Src thus promoting invadopodia activity [41]. Therefore, our results imply that inhibiting Pyk2 at invadopodia is able to counteract the effect of Src relocalisation at these sites. On the other hand, it is not clear whether FAK and Pyk2 have redundant or specific effects on FA dynamics and cell migration. As the dual FAK/Pyk2 inhibitor did not further inhibit cell migration as compared to the selective FAK inhibitor, this suggests that FAK and Pyk2 have redundant effect on melanoma cell migration. This is in accordance with previous results showing that knockdown of FAK or Pyk2 in breast cancer cells displayed similar inhibitory effect on 2D migration [41]. Thus, altogether, these results show that inhibiting a protein implicated in both invadopodia and FA biological processes have the potential to decrease both cell migration and ECM remodelling thereby reducing efficiently cancer cell invasion.

## Supplementary information

Supplemental Figure 1

Supplemental Figure 2

Supplemental Figure 3

Supplemental Figure 4

Video 1

Video 2

Video 3

Video 4

Video 5

Legend Supplemental figures and video

aj-checklist

Supplemental Table 1

## Data Availability

All datasets generated and analysed during this study are available from the corresponding author on reasonable request.
