# Supplementary figures and images for: Protein dynamics at invadopodia control invasion–migration transitions in melanoma cells
Source: Cell Death Dis. 2023 Mar 11;14(3):190. doi: 10.1038/s41419-023-05704-4 (PMC10006204; doi:10.1038/s41419-023-05704-4)

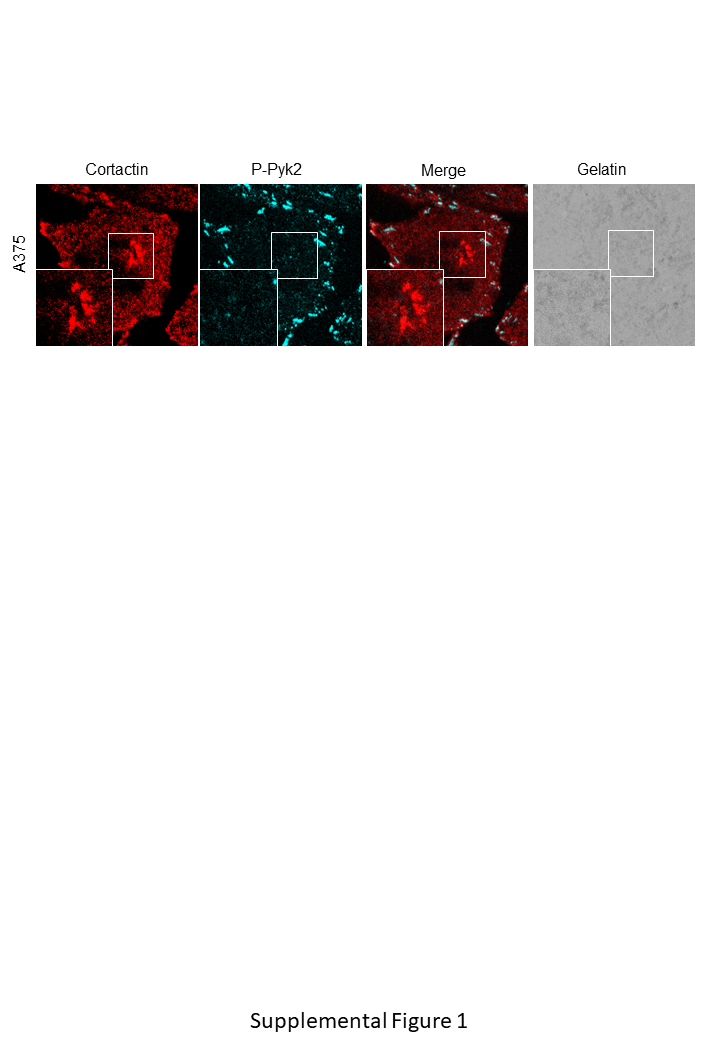

Supplement: Supplementary file 1 — Supplemental Figure 1 [file 41419_2023_5704_MOESM1_ESM.tif]

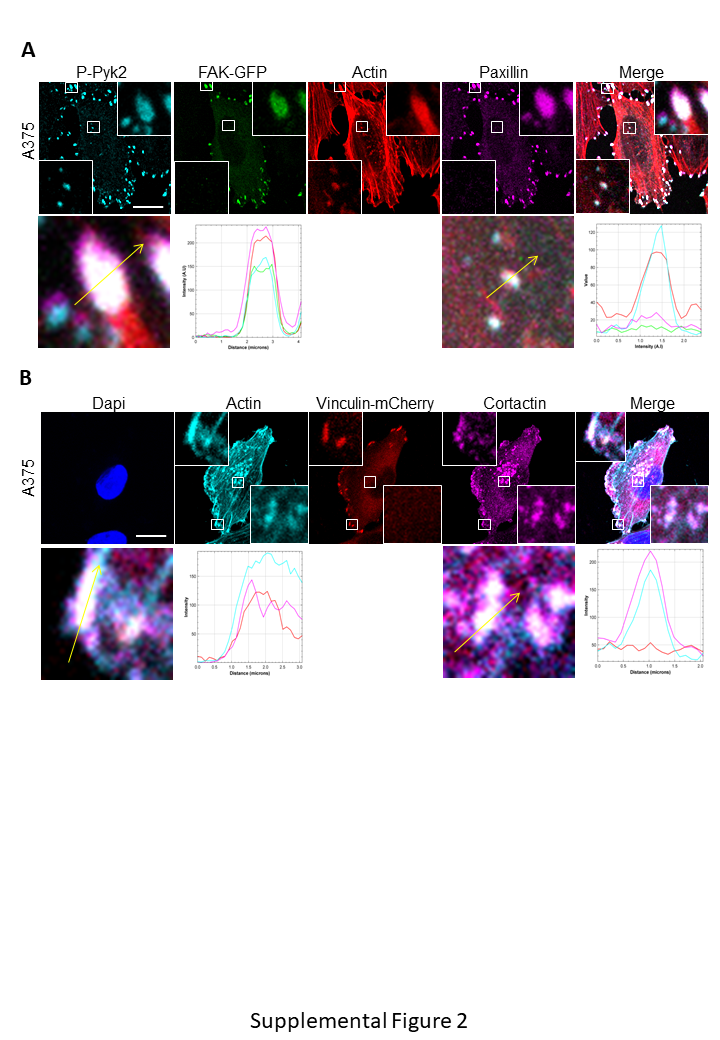

Supplement: Supplementary file 2 — Supplemental Figure 2 [file 41419_2023_5704_MOESM2_ESM.tif]

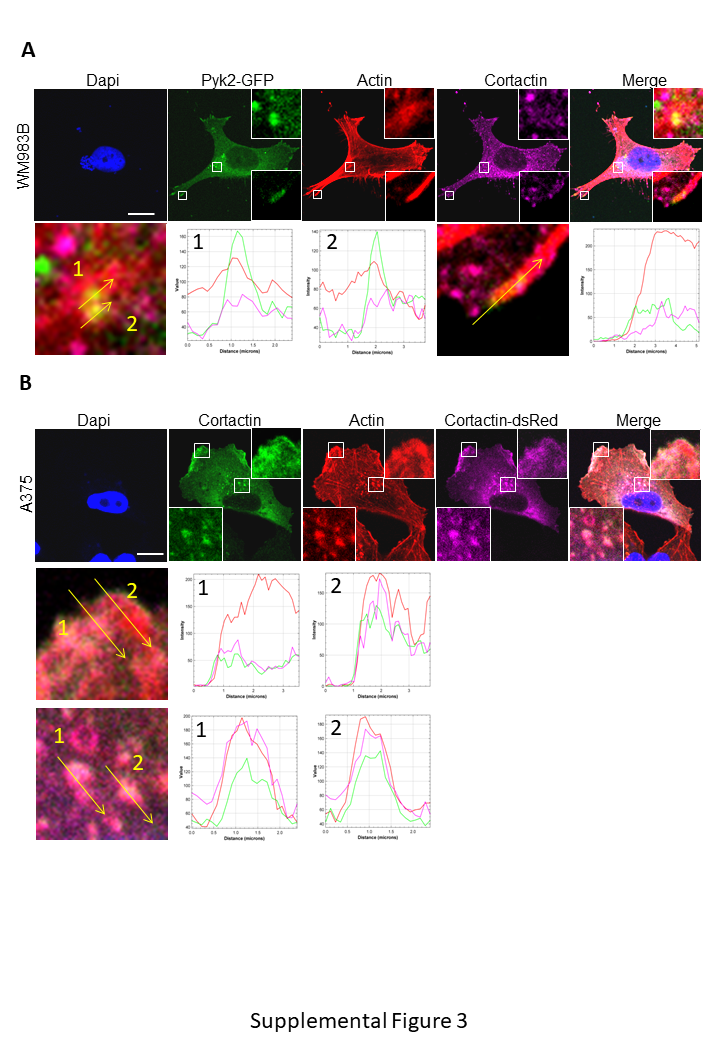

Supplement: Supplementary file 3 — Supplemental Figure 3 [file 41419_2023_5704_MOESM3_ESM.tif]

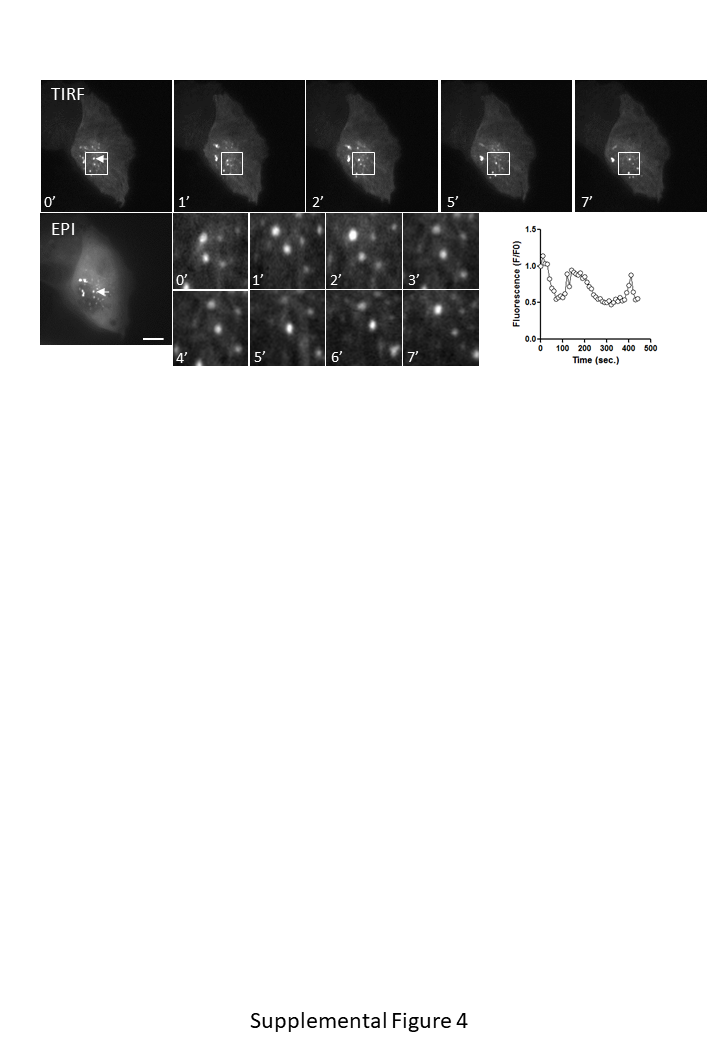

Supplement: Supplementary file 4 — Supplemental Figure 4 [file 41419_2023_5704_MOESM4_ESM.tif]

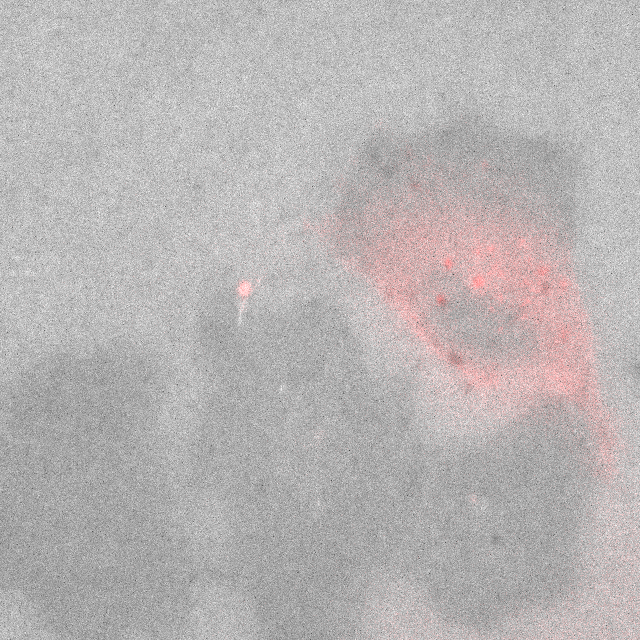

Supplement: Supplementary file 5 — Video 1 [file 41419_2023_5704_MOESM5_ESM.tif]

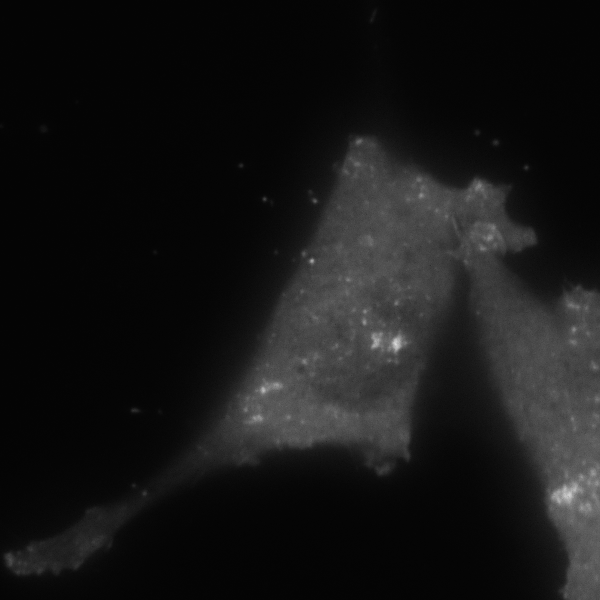

Supplement: Supplementary file 7 — Video 3 [file 41419_2023_5704_MOESM7_ESM.tif]

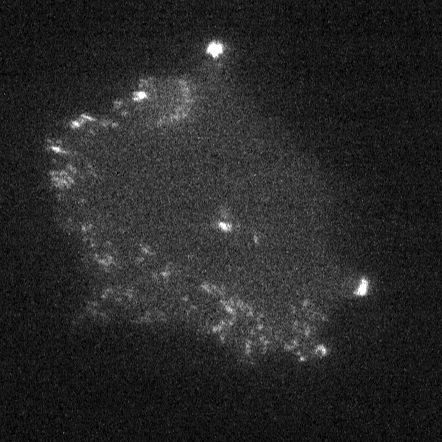

Supplement: Supplementary file 8 — Video 4 [file 41419_2023_5704_MOESM8_ESM.tif]

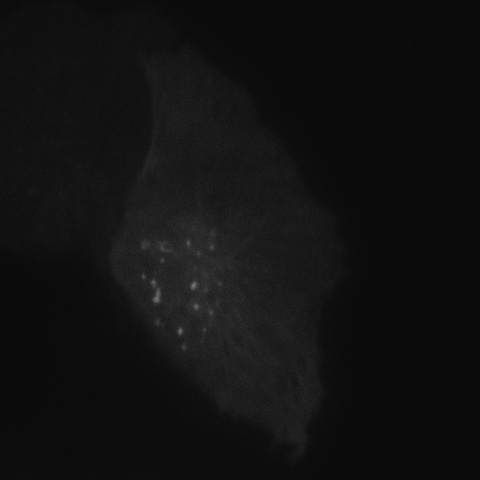

Supplement: Supplementary file 9 — Video 5 [file 41419_2023_5704_MOESM9_ESM.tif]

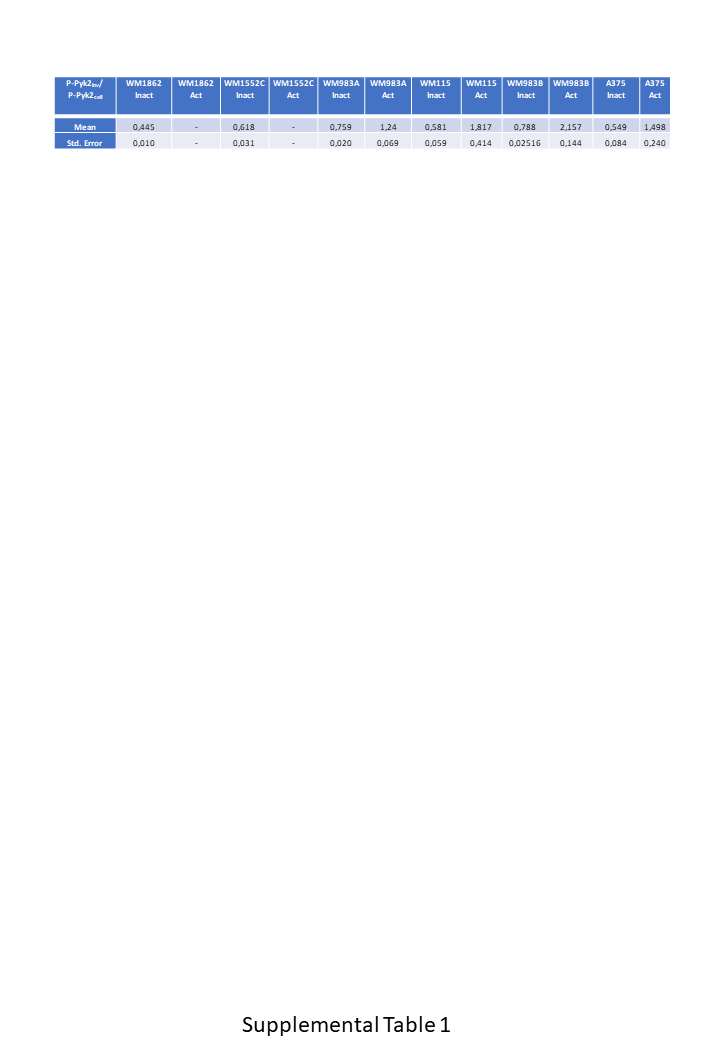

Supplement: Supplementary file 12 — Supplemental Table 1 [file 41419_2023_5704_MOESM12_ESM.tif]
