# Supplementary material for: Protein dynamics at invadopodia control invasion–migration transitions in melanoma cells
Source: Cell Death Dis. 2023 Mar 11;14(3):190. doi: 10.1038/s41419-023-05704-4 (PMC10006204; doi:10.1038/s41419-023-05704-4)
Supplement: Supplementary file 10 — Legend Supplemental figures and video [file 41419_2023_5704_MOESM10_ESM.docx]

**Supplemental Table 1: Active invadopodia displayed high level of P-Pyk2 signal.** In-situ invasive and metastatic melanoma cells were plated on FITC-Gelatin, fixed, and labelled for actin, cortactin and P-Pyk2. P-Pyk2 was quantified by normalizing P-Pyk2 signal at invadopodia over P-Pyk2 in the whole cell. Inactive invadopodia were defined as dot-like actin/cortactin with no degradation in the gelatin layer whereas active invadopodia were defined as colocalisation of actin/cortactin with degradation areas in the gelatin layer. The mean value ± SEM of P-Pyk2_invadopodia_/P-Pyk2_cell_ are presented.

**Supplemental Figure 1:** A375 melanoma cells treated with GM6001 were plated on FITC-Gelatin (Gray), fixed, and labelled for cortactin (Red) and P-Pyk2 (Cyan). Boxed regions and insets depict invadopodia (White). Note the absence of degradation at the gelatin layer and the absence of p-Pyk2 at invadopodia.

**Supplemental Figure 2: FAK-GFP and Vinculin-mCherry localize at focal adhesion but not at invadopodia. (A)** A375 melanoma cells transiently transfected with FAK-GFP were fixed and labelled for P-Pyk2 (Cyan), actin (Red) and paxillin (Magenda). Boxed regions and insets depict typical focal adhesion (White, top) and dot-like invadopodia (White, bottom). Graphs indicate fluorescent intensity in arbitrary units (A.U.) of P-Pyk2 (Cyan), FAK-GFP (Green), actin (Red) and paxillin (Magenda) over the indicated line scan in the inset. Note high fluorescence intensity of the 4 markers at focal adhesion (left) and the background level of FAK-GFP and Paxillin at invadopodia (right). Scale bar: 10 µm. **(B)** A375 melanoma cells transiently transfected with Vinculin-mCherry were fixed stained with Dapi and labelled for actin (Cyan), and cortactin (Magenda). Boxed regions and insets depict typical focal adhesion (White, top) and dot-like invadopodia (White, bottom). Graphs indicate fluorescent intensity in arbitrary units (A.U.) of actin (Cyan), vinculin (Red), and cortactin (Magenda) over the indicated line scan in the inset. Note high fluorescence intensity of actin, vinculin and cortactin at focal adhesion (left) and the background level of vinculin-mCherry at invadopodia (right). Scale bar: 10 µm.

**Supplemental Figure 3: Pyk2-GFP and Cortactin-dsRed localize to a subset of focal adhesion and invadopodia.** **(A)** WM983B melanoma cells transiently transfected with Pyk2-GFP were fixed stained with Dapi and labelled for actin (Red) and Cortactin (Magenda). Boxed regions and insets depict typical dot-like invadopodia (White, top) and focal adhesion (White, bottom). Graphs indicate fluorescent intensity in arbitrary units (A.U.) of Pyk2-GFP (Green), actin (Red) and cortactin (Magenda) over the indicated line scan in the inset. Note high fluorescence intensity of Pyk2-GFP (left) at invadopodia 1 and 2 and the presence of Pyk2-GFP at focal adhesion Scale bar: 10 µm. **(B)** A375 melanoma cells transiently transfected with Cortactin-dsRed were fixed stained with Dapi and labelled for cortactin (Green) and actin (Red). Boxed regions and insets depict membrane ruffle-containing focal adhesion (White, top) and dot-like invadopodia (White, bottom). Graphs indicate fluorescent intensity in arbitrary units (A.U.) of cortactin (Green), actin (Red), and cortactin-ds-Red (Magenda) over the indicated line scan in the inset. Note variable levels of fluorescence intensityof cortactin at focal adhesion (top) and high levels at invadopodia (bottom). Scale bar: 10 µm.

**Supplemental Figure 4: Analysis of invadopodia dynamics and Tks5 dynamics at invadopodia.** A375 cells expressing Tks5-GFP were imaged by dual Epifluorescence/TIRF mode at 1 image/10 sec during 1 hour. Insets show magnified views of invadopodia (arrows). One image in epifluorescence (EPI) is shown for each condition to help visualise the cortical location of invadopodia. Note the high dynamic of Tks5 (C) Graph represent normalized fluorescence intensity over time at invadopodia and nascent adhesions. Scale bar: 10 µm.

**Video 1**: Dual time-lapse imaging (1 image/15 min) of A375 cells expressing cortactin-dsRed and plated on Cy3-Gelatin.

**Video 2**: Time-lapse imaging (1 image/15 min) of A375 cells expressing cortactin-dsRed plated on FITC-Gelatin. Wide field (top), cortactin (middle) and gelatin (bottm) fluorescence images are shown.

**Video 3**: Time-lapse imaging (1 image/10 sec in Azimuthal TIRF mode) of A375 cells expressing cortactin-dsRed.

**Video 4**: Time-lapse imaging (1 image/10 sec in Azimuthal TIRF mode) of A375 cells expressing Pyk2-GFP.

**Video 5:** Time-lapse imaging (1 image/10 sec in Azimuthal TIRF mode) of A375 cells expressing Tks5-GFP.
